
JOURNAL INFORMATION
==============================
NLM Title Abbreviation: Behav Neurol
Journal ID: Behav Neurol
NLM Journal ID: 8914585
Journal ID: BN

Behavioural Neurology

ISSN: 0953-4180
EISSN: 1875-8584
Publisher: Wiley

ARTICLE INFORMATION
==============================
PMCID: PMC5434382
PMID: 21422553
DOI: 10.3233/BEN-2010-0293
Article ID: 409306
Article version: 1
Subjects: Research Article

Recovering from Acquired Childhood Aphasia (ACA)–20 Years Later, Learning about the Neuroplasticity of Language

Lauterbach Martin 1 *
Gil da Costa Ricardo 2
Leal Gabriela 3
Willmes Klaus 4
Martins Isabel Pavão 1
1 Language Laboratory Institute of Molecular Medicine University of Lisbon Lisbon Portugal
2 Salk Institute San Diego CA USA
3 Santa Maria Hospital Lisbon Portugal
4 Section Neuropsychology University Hospital RWTH Aachen University Aachen Germany
*Martin Lauterbach: mlauterbach@fm.ul.pt
Print publication date: 2010
Electronic publication date: 2011 Mar 7
Volume: 23
Issue: 4
First page: 195
Last page: 197
Received 2011 Jan 31; Accepted 2011 Jan 31
Copyright: Copyright © 2010 Hindawi Publishing Corporation and the authors.
Copyright year: 2010
License: This is an open access article distributed under the Creative Commons Attribution License, which permits unrestricted use, distribution, and reproduction in any medium, provided the original work is properly cited.
License URL: https://creativecommons.org/licenses/by/3.0/

==============================
Behavioural Neurology 23 (2010) 195–197
DOI 10.3233/BEN-2010-0293
IOS Press

### Recovering from acquired childhood aphasia (ACA) – 20 years later, learning about the neuroplasticity of language

Martin Lauterbach[a,*], Ricardo Gil da Costa[b], Gabriela Leal[c], Klaus Willmes[d] and Isabel Pavão Martins[a]
[a]*Language Laboratory, Institute of Molecular Medicine, University of Lisbon, Lisbon, Portugal*
[b]*Salk Institute, San Diego, CA, USA*
[c]*Santa Maria Hospital, Lisbon, Portugal*
[d]*Section Neuropsychology, University Hospital, RWTH Aachen University, Aachen, Germany*

### 1. Introduction

Language is at the core of human cognitive abilities and the mechanisms associated with neural recovery from aphasia are still largely unknown. Important insights have been reported, such as: i) Significantly better recovery occurs, if the brain lesions leading to aphasia were suffered during childhood (ACA); ii) that *perilesional map expansion* and *transference of language functions to homologue areas in the contralateral hemisphere* are thought to be two main processes sustaining this neuroplasticity [1]. From the study of peri-natal brain lesions of left hemisphere is known that they are often associated with language transfer to the right hemisphere [2–4].

We investigated if potential factors underlying the neuroplasticity of language (such as the developmental linguistic stage at onset of aphasia and size of the lesion) would significantly contribute to determine the recovery strategy. We report the a follow-up study of three cases of left frontal subcortical isquemic strokes about 20 years after onset of aphasia.

### 2. Method

### *2.1. Participants*

Three young adults (1 female DF, 2 male JAF + JPF) that suffered from a aphasia, due to a left frontal subcortical lesion (age at onset JAF 2,1; DF 2,7; JPF 13,5 years respectively), performed an extensive neurolinguistic testing (age at testing 31,5; 33,4; 26,3 years respectively) applying 20 different neurolinguistic tests.

The activation pattern of the 3 patients were compared to 4 (3 female, 1 male) healthy controls (mean age of 26,3 years and 15 years of formal education).

### *2.2. MRI*

MRI data acquisition was performed on 3T Philips Achieva Scanner. Two fMRI language paradigms, semantic decision paradigm with both visual and auditory presentation were applied. Visual semantic decision paradigm, written in block design; image acquisition was performed in a continuous manner (TR 2000 ms, 160 volumes). Auditory semantic decision paradigm, written in event design; image acquisition was performed in sparse design, in order to present the auditory stimulus without interference of the scanner noise (TR 8000 ms, 120 volumes). Functional data was processed using the BrainVoyager software. Single and group General Linear Models (GLM) were applied to the data. For the visual paradigm activation clusters were corrected for multiple comparisons after the Bonferroni method to an p-level of 0,05. The threshold for the auditory paradigm was chosen less strictly (auditory stimuli $p <$ 0,000059, without correction for multiple comparisons), due to the weaker signal of the data of the event related paradigm. For anatomical localization of activation clusters peak-voxel cluster were defined

*Corresponding author. E-mail: mlauterbach@fm.ul.pt.

ISSN 0953-4180/10/$27.50 © 2010 – IOS Press and the authors. All rights reserved

Table 1

**SEMANTIC DECISION visual** stimuli p (Bonf) $< 0.05$

| | Control | DF | JPF | JAF |
|---|---|---|---|---|
| cluster 1 | LEFT BA4 | LEFT BA8 | LEFT BA3 | RIGHT BA 22 |
| cluster 2 | LEFT BA6 | LEFT BA6 | LEFT BA40 | RIGHT BA 46 |
| cluster 3 | LEFT BA9 | LEFT BA6 | LEFT BA45 | RIGHT BA 6 |
| cluster4 | LEFT BA22 | LEFT BA9 | LEFT BA21 | RIGHT BA10 |
| cluster 5 | LEFT BA45 | LEFT BA39 | LEFT BA21 | RIGHT BA 6 |
| cluster 6 | | LEFT BA21 | LEFT BA 40 | LEFT BA 9 |
| cluster 7 | | LEFT BA3 | | LEFT CEREBELLUM |
| cluster 8 | | LEFT BA21 | | LEFT BA 22 |

**SEMANTIC DECISION** auditory stimuli $p < 0{,}000059$

| | Control | DF | JPF | JAF |
|---|---|---|---|---|
| cluster 1 | LEFT BA21 | LEFT BA13 | LEFT BA39 | RIGHT BA32 |
| cluster 2 | LEFT BA22 | LEFT BA 22 | LEFT BA40 | RIGHT BA9 |
| cluster 3 | LEFT BA 41 | | LEFT BA22 | RIGHT BA24 |
| cluster 4 | | | LEFT BA43 | LEFT BA24 |
| cluster 5 | | | | LEFT BA32 |
| cluster 6 | | | | LEFT BA20 |
| cluster 7 | | | | LEFT BA20 |
| cluster 8 | | | | LEFT BA39 |
| cluster 9 | | | | LEFT BA 22 |
| cluster 10 | | | | LEFT BA42 |
| cluster 11 | | | | LEFT BA21 |

with a cluster threshold of 300 voxels for the visual condition and 150 voxels for the auditory condition. All participants performed a T1-weighted anatomical scan. All anatomical data were transformed into the common space of Talairach.

### 3. Results

In all 3 patients structural MRI showed the sequelae of a left frontal subcortical infarction in a similar localization but of different size. All three patients showed fully functional recovery of language; however JAF performed below one SD in the semantic (food and animals) and the phonological (letters M,P,R) verbal fluency task. JAF, but also JPF, admitted, as the only symptom, some word finding difficulties in the self evaluation form. In Table 1 the respective Brodmann areas of the peak-voxel VOIs are listed. The main pattern of recovery in patients DF and JPF was reorganization of language function in the dominant left hemisphere. The exception is patient JAF who had a partial language transfer. His neuronal network for language is much larger distributed (e.g. involvement of the left cerebellum). The homologue language areas (BA22 and BA46) of the right hemisphere are more activated by the visual stimuli of the language paradigm than their left counterparts, whereas heard language stimuli continue to be processed predominantly in the left hemisphere.

### 4. Conclusion

The results contrasts to what is known from perinatal brain lesions before language acquisition that are often associated with a complete language shift to the right hemisphere [4]. This suggests that an early commitment of the left hemisphere to language initiates an almost irreversible specialization [5–7]. Only when the left hemisphere is compromised by a sufficient large lesion language functions that are acquired later on tend to be transferred to the homologue areas of the contra-lateral hemisphere. In JAFs case the processing of written stimuli (acquired 4 years after lesion onset) was directed to the right hemisphere. Further research should investigate the underlying factors in the exceptional cases of transference of language function to the opposite brain hemisphere.

### References

[1] D. Saur, R. Lange, A. Baumgaertner, V. Schraknepper, K. Willmes, M. Rijntjes and C. Weiller, Dynamics of language reorganization after stroke, *Brain* (2006), 1371–1384.

[2] J.M. Tillema, A.W. Byars, L.M. Jacola, M.B. Schapiro, V.J. Schmithorst, J.P. Szaflarski and S.C. Holland, Cortical reorganization of language functioning following perinatal left MCA stroke, *Brain and Language* (2008), 184–194.

[3] F. Liégeois, A. Connelly, J. Cross, S.G. Boyd, D.G. Gadian, F. Vargha-Khadem and T. Baldeweg, Language reorganization in children with early onset lesions of the left hemisphere: an fMRI study, *Brain* (2004), 1229–1236.

[4] M. Staudt, K. Lidzba, W. Grodd, D. Wildgruber, M. Erb and I. Krägeloh-Mann, Right-Hemispheric Organization of Language Following Early Left-Sided Brain Lesions: Functional MRI Topography, *NeuroImage* (2002), 954–967.

[5] D.P. Anderson, A.S. Harvey, M.M. Saling, V. Anderson, M. Kean, D.F. Abbott, R.M. Wellard and G.D. Jackson, fMRI Lateralization of Expressive Language in Children with Cerebral Lesions, *Epilepsia* (2006), 998–1008.

[6] L.A. Balsamo, B. Xu, C.B. Grandin, J. Petrella, S.H. Braniecki, T.K. Elliott and W.D. Gaillard, A Functional Magnetic Resonance Imaging Study of Left Hemisphere Language Dominance in Children, *Arch Neurol* (2002), 1168–1174.

[7] I.P. Martins, C. Loureiro, S. Ramos and T. Moreno, Grammatical dissociation during acquired childhood aphasia. Developmental Medicine and Child Neurology (2009).
